# Neutrophil Depletion Exacerbates Pregnancy Complications, Including Placental Damage, Induced by Silica Nanoparticles in Mice

**DOI:** 10.3389/fimmu.2018.01850

**Published:** 2018-08-08

**Authors:** Kazuma Higashisaka, Akitoshi Nakashima, Yuki Iwahara, Aiko Aoki, Masahiro Nakayama, Itaru Yanagihara, Ying Lin, Kazuya Nagano, Shin-ichi Tsunoda, Shigeru Saito, Yasuo Yoshioka, Yasuo Tsutsumi

**Affiliations:** ^1^Laboratory of Toxicology and Safety Science, Graduate School of Pharmaceutical Sciences, Osaka University, Suita, Japan; ^2^Department of Legal Medicine, Osaka University Graduate School of Medicine, Suita, Japan; ^3^Department of Obstetrics and Gynecology, University of Toyama, Toyama, Japan; ^4^Department of Developmental Medicine, Research Institute, Osaka Women’s and Children’s Hospital, Izumi, Japan; ^5^The Faculty of Pharmaceutical Sciences, Kobe Gakuin University, Kobe, Japan; ^6^Laboratory of Biopharmaceutical Research, National Institutes of Biomedical Innovation, Health and Nutrition, Ibaraki, Japan; ^7^The Center for Advanced Medical Engineering and Informatics, Osaka University, Suita, Japan; ^8^Vaccine Creation Project, BIKEN Innovative Vaccine Research Alliance Laboratories, Research Institute for Microbial Diseases, Osaka University, Suita, Japan; ^9^BIKEN Center for Innovative Vaccine Research and Development, The Research Foundation for Microbial Diseases of Osaka University, Suita, Japan

**Keywords:** apoptosis, nanotoxicology, placenta, placental vessels, pregnancy complications

## Abstract

Recent advances in nanotechnology have led to the development of nanoparticles with innovative functions in various fields. However, the biological effects of nanoparticles—particularly those on the fetus—need to be investigated in detail, because several previous studies have shown that various nanoparticles induce pregnancy complications in mice. In this regard, our previous findings in mice suggested that the increase in peripheral neutrophil count induced by treatment with silica nanoparticles with a diameter of 70 nm (nSP70) may play a role in the associated pregnancy complications. Therefore, here, we sought to define the role of neutrophils in nSP70-induced pregnancy complications. The peripheral neutrophil count in pregnant BALB/c mice at 24 h after treatment with nSP70 was significantly higher than in saline-treated mice. In addition, maternal body weight, uterine weight, and the number of fetuses in nSP70-treated mice pretreated with anti-antibodies, which deplete neutrophils, were significantly lower than those in nSP70-treated mice pretreated with phosphate-buffered saline or isotype-matched control antibodies. Histology revealed that neutrophil depletion increased nSP70-induced placental damage from the decidua through the spongiotrophoblast layer and narrowed spiral arteries in the placentae. In addition, depletion of neutrophils augmented nSP70-induced cytotoxicity to fetal vessels, which were covered with endothelium. The rate of apoptotic cell death was significantly higher in the placentae of anti-nSP70-treated mice than in those from mice pretreated with isotype-matched control antibodies. Therefore, impairment of placental vessels and apoptotic cell death due to nSP70 exposure is exacerbated in the placentae of nSP70-treated mice pretreated with anti-antibodies. Depletion of neutrophils worsens nSP70-induced pregnancy complications in mice; this exacerbation was due to enhanced impairment of placental vessels and increased apoptotic cell death in maternal placentae. Our results provide basic information regarding the mechanism underlying silica-nanoparticle-induced pregnancy complications.

## Introduction

Compared with conventional materials, nanoparticles offer unique physicochemical properties and innovative functions. Consequently, research into nanoparticles and their development and commercialization in various industrial fields, such as food, cosmetics, and medicine, is rapidly progressing (1–3). However, one bottleneck in the development of nanoparticles is that their size-associated novel functions have the potential to exert unknown biological effects at unexpected sites in the body. Therefore, the expanding use of nanoparticles has increased the urgency of collecting relevant safety information. In particular, the reproductive toxicity of chemical substances is a public health concern. Because infants typically are more sensitive to environmental toxins than adults (4), infants and fetuses may experience unexpected effects even when they are exposed to amounts that are nontoxic to adults. Several recent studies have reported on the reproductive toxicity of nanoparticles (5–7). For example, we previously demonstrated that intravenous treatment with silica nanoparticles 70 nm in diameter (nSP70) induced greater intrauterine growth restriction and placental damage in mice than did silica particles larger than 100 nm (5). We speculated that nSP70-induced pregnancy complications were due to placental damage, which did not occur in the mice treated with larger particles (5). However, details of the mechanism underlying this effect are minimally understood.

To address this insufficiency, we focused on the roles of neutrophils, which are the most abundant leukocytes in humans and important factors in placental dysfunction and direct damage to developing embryos (8, 9). For example, increased neutrophils counts promoted endothelial dysfunction after placental ischemia (10), and endothelial cell damage subsequent to neutrophil activation may contribute to preeclampsia and intrauterine growth restriction (11, 12). Moreover, neutrophils are associated with vascular dysfunction in preeclamptic women, and activated neutrophils may induce increased production of myeloperoxidase in the placental and endothelial cells of these patients (13, 14). In fact, we previously showed that intravenous treatment with nSP70 increased peripheral neutrophil counts in nonpregnant mice (15). Therefore, we consider that neutrophils might be components of the mechanism by which nSP70 induce pregnancy complications.

Here, we evaluated the role of neutrophils in nSP70-associated pregnancy complications in mice. Our results demonstrate that neutrophils may protect against pregnancy complications—especially the nSP70-triggered breakdown of pregnancy maintenance. Our results provide important information regarding the mechanism underlying nSP70-induced pregnancy complications.

## Materials and Methods

### Animals

Pregnant BALB/c mice [age, 8–10 weeks; gestational day (GD) 13–14] were purchased from Nippon SLC (Shizuoka, Japan). The mice were housed in a ventilated animal room maintained at 20 ± 2°C with a12:12-h light:dark cycle and given unrestricted access to water and forage (FR-2, Funabashi Farm, Chiba, Japan). Dams were weighed daily.

### Silica Nanoparticles

Silica nanoparticles were purchased from Micromod Partikeltechnologie (Rostock–Warnemünde, Germany). Before use, the particles were sonicated for 5 min and vortexed for 1 min. Preparations of silica nanoparticles were checked for contamination with lipopolysaccharide by using an LAL Endotoxin Assay Kit (GenScript, Piscataway, NJ, USA).

### Injection of Silica Nanoparticles

Pregnant BALB/c mice were injected intravenously with nSP70 (0.8 mg/mouse) on GD 16 and then euthanized under anesthesia on GD 17. Blood samples collected at 24 h after treatment and before euthanasia were centrifuged at 3,000 *g* for 15 min to obtain plasma. Uteri, fetuses, and placentae were weighed, and the placentae were prepared for histological examination.

### *In Vivo* Neutrophil Depletion

Neutrophil depletion was achieved by intraperitoneal injection of anti-Ly-6G antibodies (clone 1A8; BioLegend, San Diego, CA, USA), isotype-matched control antibodies (clone RTK2758; BioLegend), or phosphate-buffered saline (PBS) into pregnant BALB/c mice (*n* = 5 or 6 per group) at 24 h before nSP70 injection (that is, on GD 15).

### Flow Cytometry

Red blood cells in collected blood samples were lysed with ammonium chloride. All staining procedures were performed in PBS containing 2% fetal calf serum. To minimize nonspecific binding, we preincubated single-cell suspensions with anti-CD16/CD32 antibodies (clone 93; eBioscience, San Diego, CA, USA). Cells were labeled with combinations of phycoerythrin-conjugated Gr-1 antibodies (clone RB6-8C5; eBioscience), allophycocyanin-conjugated CD11b antibodies (clone M1/70; BD Pharmingen, San Diego, CA, USA), fluorescein-isothiocyanate-conjugated F4/80 antibodies (clone CI:A3-1; AbD Serotec, Oxford, UK), and phycoerythrin-Cy7-conjugated CD11c antibodies (clone HL3; BD Pharmingen). The cells were resuspended in staining buffer containing 7-amino-actinomycin D (BD Pharmingen), and the stained cells were analyzed for surface phenotype by means of a FACS Canto flow cytometer (BD Biosciences, Franklin Lakes, NJ, USA). The cells were gated according to side-scattered light (SSC) area and forward-scattered light (FSC) area and then according to SSC height/SSC width, FSC height/FSC width, and 7-amino-actinomycin D staining to eliminate doublet cells and dead cells (parent population). The proportion of neutrophils (that is, CD11b^+^ Gr-1^+^ F4/80^−^) was calculated as a percentage of the parent population.

### Histology

At 24 h after the administration of nSP70, placentae were removed from mice and placed in fixative solution (10% neutral buffered formalin, Nacalai Tesque, Kyoto, Japan). Paraffin-embedded sections were prepared and stained by the Applied Medical Research Laboratory (Osaka, Japan).

### Immunohistochemistry

At 24 h after the administration of nSP70, the placentae of mice were removed and placed in fixative solution (10% neutral buffered formalin). Sections (thickness, 5 µm) were deparaffinized in xylene, rehydrated in a graded series of alcohol, and boiled in citrate buffer at 121°C for 15 min in an autoclave for antigen retrieval. Endogenous peroxidase activity was quenched by incubating in 3% hydrogen peroxide in methanol for 15 min, and the sections were then incubated in 5% normal goat serum to block nonspecific binding. After extensive washes with PBS, the sections were reacted with anti-CD31 (clone ab124432; Abcam, Cambridge, MA, USA) as a primary antibody and the detection was performed according to the manufacturer’s instructions provided with the Vectastain kit (Vector Laboratories, Burlingame, CA, USA).

### Measurement of the Areas of Fetal and Maternal Vessels

To precisely differentiate fetal vessels from maternal vessels in the labyrinth layer of placentae, we performed CD31 staining; fetal vessels and sporadic nucleated erythrocytes are CD31-positive, whereas maternal vessels are CD31-negative (16) (Figure S1 in Supplementary Material). Three random areas were selected per placenta, and the proportions of fetal and maternal vessel areas to total area were calculated by using ImageJ software (National Institutes of Health, Bethesda, MD, USA). At least five placentas in each group were evaluated.

### Evaluation of Apoptosis in Placentae

Transferase-mediated dUTP nick-end labeling (TUNEL) staining (catalog no. G7130, DeadEnd Colorimetric TUNEL System, Promega, Fitchburg, WI, USA) was used to detect apoptotic cells. Placental tissue sections were prepared according to the manufacturer’s instructions (Vectastain Kit, Vector Laboratories). After being washed, sections were counterstained with Mayer’s hematoxylin, washed in water, and successively immersed in graded ethanol solutions and xylene before being cover slipped. In control sections, control nonimmune mouse IgG (Vector Laboratories) was used as the primary antibody. TUNEL-positive nuclei (apoptotic nuclei) in the placenta were counted, and the apoptotic index of each section was calculated as the number of nuclei that stained TUNEL-positive as a percentage of the total number of nuclei found within the section.

### Statistical Analyses

**Figure 1:**
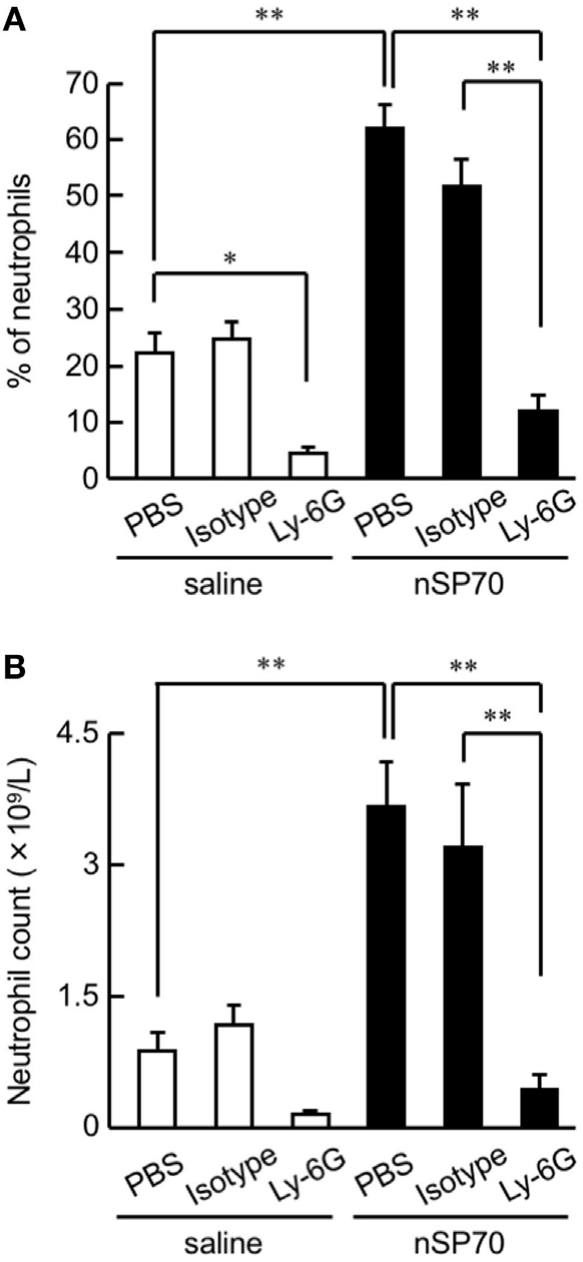
Effects of treatment with silica nanoparticles on neutrophil levels in pregnant mice. Pregnant BALB/c mice were intravenously injected with silica nanoparticles with a diameter of 70 nm (0.8 mg/mouse) or saline on gestational day 16. **(A)** The proportion (%) of neutrophils in the peripheral blood of each mouse was determined by flow cytometry at 24 h after treatment. **(B)** The neutrophil count in the peripheral blood of each mouse was calculated by multiplying the neutrophil proportion by the total white blood cell count. Data are presented as means ± SEM; *n* = 5 or 6; *P < 0.05 and **P < 0.01.

**Figure 2:**
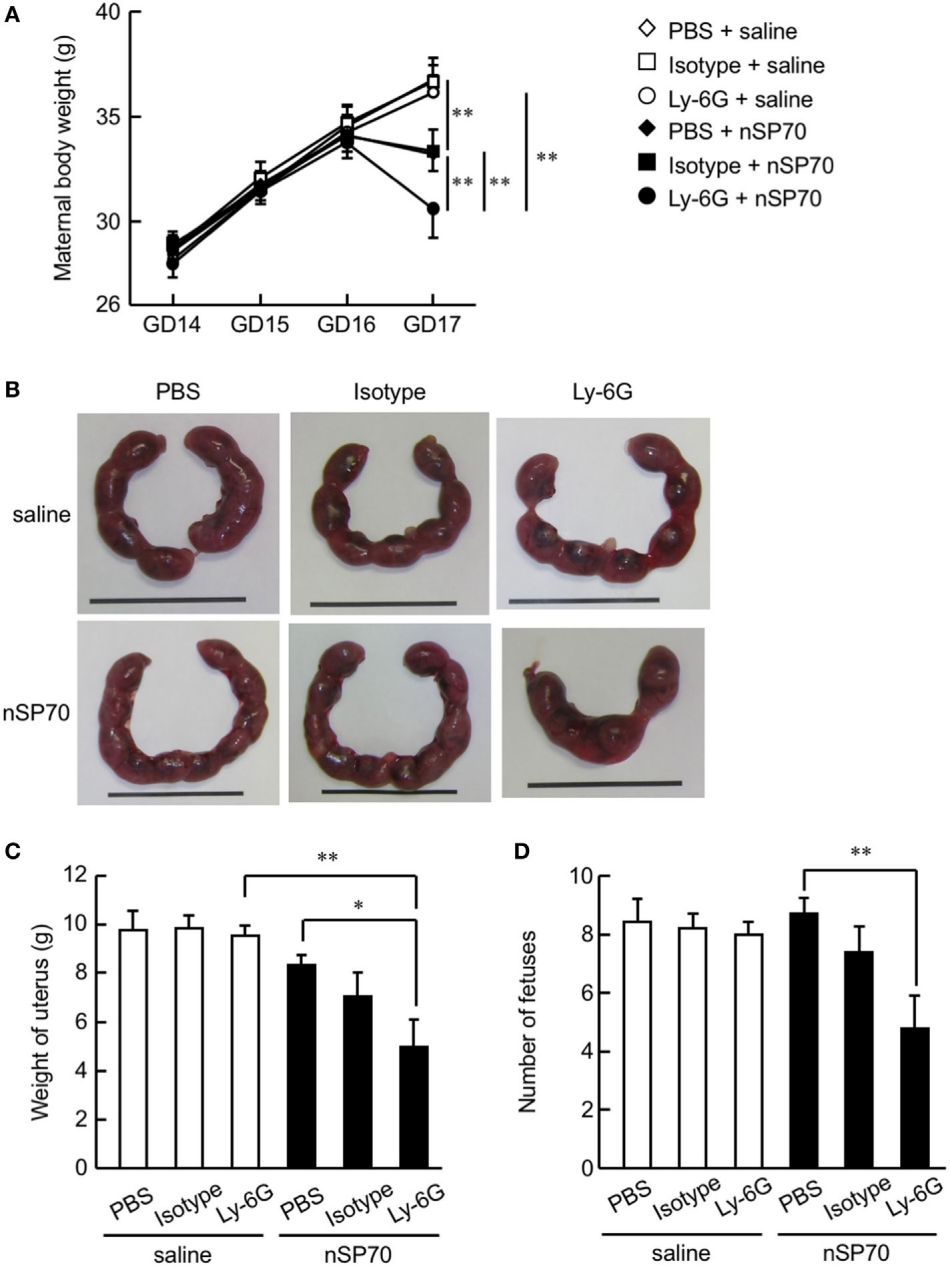
Depletion of neutrophils exacerbates the decrease in maternal body weight in silica nanoparticles with a diameter of 70 nm (nSP70)-treated mice. Pregnant BALB/c mice were intraperitoneally treated with anti-Ly-6G antibodies or isotype control antibodies (150 μg/mouse) on gestational day 15; 24 h later, they received nSP70 (0.8 mg/mouse) or saline by intravenous injection. **(A)** Maternal body weights were assessed daily. **(B)** Representative uteri from mice. On gestation day 17, **(C)** the excised uteri were weighed and **(D)** the fetuses excised from each uterus were counted. Data are presented as means ± SEM; *n* = 9 or 10; *P < 0.05 and **P < 0.01.

**Figure 3:**
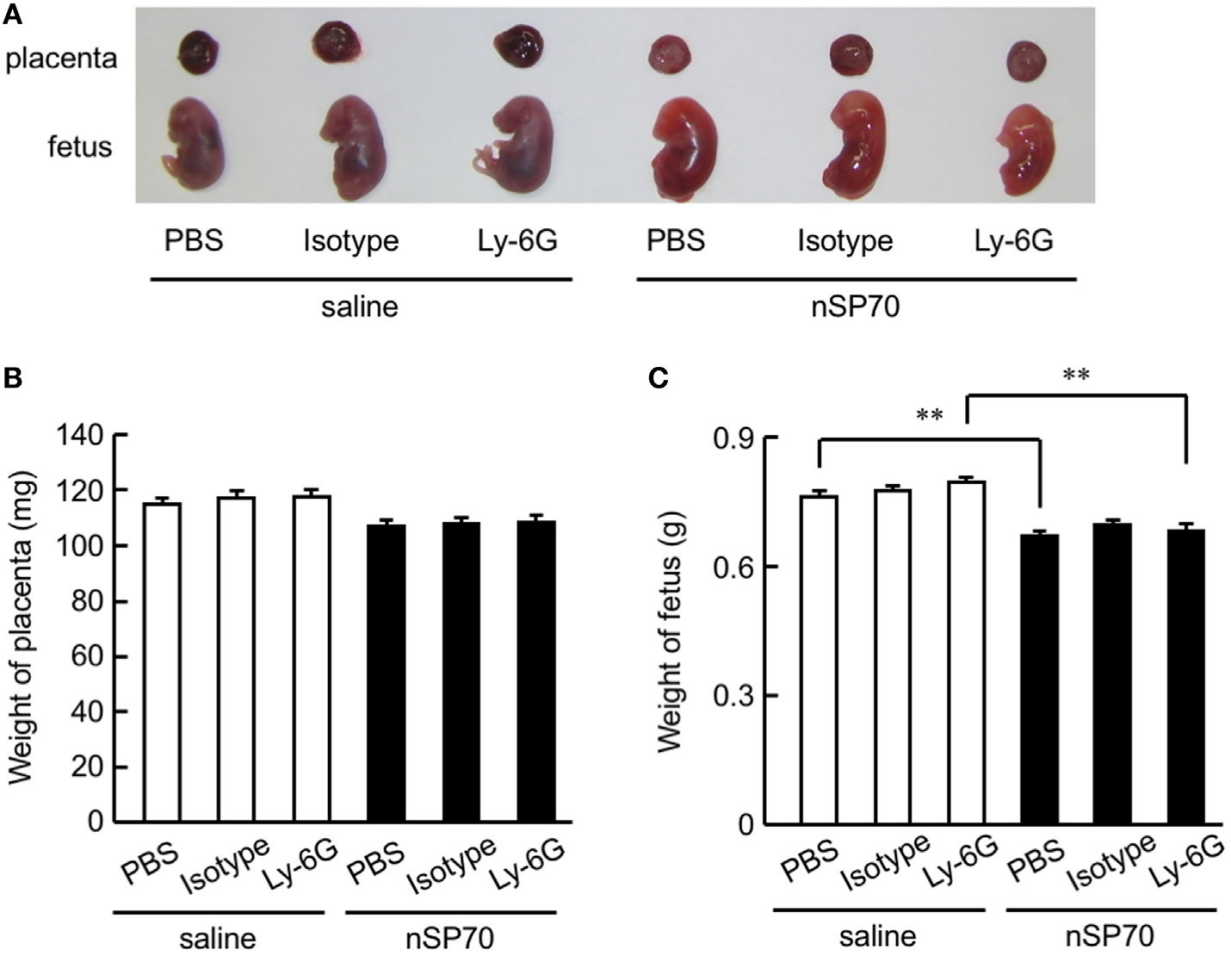
Effects of neutrophil depletion on pregnancy complications in silica nanoparticles with a diameter of 70 nm (nSP70)-treated mice. Pregnant BALB/c mice were treated intraperitoneally with anti-Ly-6G or isotype-matched control antibodies (150 μg/mouse) on gestational day (GD) 15; 24 h later, mice received either nSP70 or saline by intravenous injection. The uteri were excised on GD 17. **(A)** Representative placentae and fetuses. **(B)** Placental weight and **(C)** fetal weight were assessed. Data are presented as means ± SEM; *n* = 9 or 10; **P < 0.01.

Statistical analyses were performed by using Ekuseru–Toukei 2012 software (Social Survey Research Information Co., Ltd., Tokyo, Japan). Results shown in Figures 1–3 are expressed as means ± SEM, and Bonferroni’s method was used to compare differences. Data in the remaining figures are expressed as means ± SD, and Kruskal–Wallis and Mann–Whitney methods were used to compare differences. *P-*values lower than 0.05 were considered statistically significant.

## Results

### Induction of Neutrophilia in nSP70-Treated Pregnant Mice

To assess the proportion of neutrophils after treatment with nSP70, pregnant BALB/c mice were intravenously treated with nSP70 (0.8 mg/mouse) on GD 16; lipopolysaccharide contamination in the nSP70 solution was below the limit of detection (<0.01 EU/mL). At 24 h after treatment, the proportion of neutrophils was significantly higher in mice treated with nSP70 than in saline-treated mice, suggesting that nSP70 might also induce neutrophilia in pregnant mice (Figure 1A). To reduce the neutrophil count, we then intraperitoneally injected pregnant BALB/c mice on GD 15 with anti-Ly-6G antibodies (or PBS or isotype-matched antibodies, as controls); 24 h later, we injected them with nSP70 (0.8 mg/mouse). In nSP70-treated mice, the proportion of neutrophils was significantly lower in those pretreated with anti-Ly-6G antibodies than with PBS or isotype-matched control antibodies (Figure 1A). By multiplying the proportion of neutrophils by the total white blood cell count, we showed that pretreatment with anti-Ly-6G antibodies significantly curtailed the nSP70-induced increase in neutrophil count, thus supporting the results of the flow cytometric analysis (Figure 1B). Furthermore, we tested for the presence of different neutrophil subsets on the basis of CD16/CD62L expression. During acute inflammation, three neutrophil subsets are found in the blood: CD16^bright^/CD62L^dim^ cells that are capable of suppressing T-cell proliferation (activated); CD16^dim^/CD62L^bright^ cells with a banded nuclear morphology (immature); and phenotypically normal CD16^bright^/CD62L^bright^ neutrophils (mature) (17–19). We found that the mature neutrophil population was greater in nSP70-injected mice pretreated with PBS than in saline-injected mice pretreated with PBS, but there was no significant difference in the activated neutrophil population between these two groups (Figure S2 in Supplementary Material). This result suggests that nSP70 triggers an increase in neutrophil maturation only. In an analysis of the influence of nSP70 on other blood cell types, granulocyte counts were significantly increased in nSP70-treated mice compared with controls, and pretreatment with anti-Ly-6G antibodies significantly dampened the nSP70-induced granulocyte increase, consistent with the results of flow cytometry (Figure S3A in Supplementary Material). In contrast, the numbers of total leukocytes (Figure S3B in Supplementary Material), monocytes (Figure S3C in Supplementary Material), lymphocytes (Figure S3D in Supplementary Material), platelets (Figure S3E in Supplementary Material), and erythrocytes (Figure S3F in Supplementary Material) did not differ between nSP70-treated and control mice, regardless of anti-Ly-6G treatment. These findings suggest that the increased neutrophil count in nSP70-treated mice reflected an increase in the number of granulocytes after nSP70 injection. Moreover, the percentage of CD4^+^ T-cells in the peripheral blood was significantly lower in nSP70-injected mice pretreated with PBS than in saline-injected mice pretreated with PBS (Figure S4A in Supplementary Material), although the percentage of CD4^+^ FOXP3^+^ regulatory T-cells did not differ between nSP70-treated mice pretreated with PBS and saline-treated mice pretreated with PBS (Figure S4B in Supplementary Material). These results suggest that the proportion of T-cells decreases as the proportion of neutrophils increases in the peripheral blood of mice treated with nSP70.

### Neutrophil Depletion Exacerbates Pregnancy Complications in nSP70-Treated Mice

To evaluate the association between nSP70-induced pregnancy complications and the increased number of neutrophils after treatment with nSP70, we evaluated maternal body weight and placental weight after neutrophil depletion. Maternal body weight on GD 17 was lower in nSP70-treated mice than in saline-treated mice, as previously reported (Figure 2A) (5). Furthermore, body weight was significantly lower in nSP70-treated dams pretreated with anti-Ly-6G antibodies than in nSP70-treated mice pretreated with PBS or isotype-matched control antibodies (Figure 2A). Uteri were harvested at GD 17 (Figure 2B), and fetuses were counted. As seen with maternal body weight, pretreatment with anti-Ly-6G antibodies exacerbated the decrease in uterine weight in nSP70-treated mice compared with that after PBS (Figure 2C). Similarly, anti-Ly-6G–nSP70-treated mice had significantly fewer fetuses than PBS–nSP70-treated dams (Figure 2D). These results suggest that neutrophil depletion exacerbated nSP70-induced pregnancy complications.

We then weighed the harvested placentae and fetuses (Figure 3A). Whereas mice that had received nSP70 had smaller fetuses than those that had received saline (5), neither placental weight (Figure 3B) nor number of fetuses (Figure 3C) differed between nSP70-injected mice pretreated with anti-Ly-6G antibodies and those given PBS or isotype-matched control antibodies. Next, to assess the viability of pups born to nSP70-treated mice after neutrophil depletion, pregnant BALB/c mice were intraperitoneally injected with anti-Ly-6G or isotype control-matched antibodies 24 h before intravenous injection of nSP70 (0.8 mg/mouse). Whereas the number of neonates did not differ between nSP70-treated mice pretreated with PBS or saline (Figure S5A in Supplementary Material), nSP70-treated mice pretreated with anti-Ly-6G antibodies tended to birth fewer live pups than those pretreated with PBS or isotype-matched control antibodies. In comparison, the body weight (Figure S5B in Supplementary Material) and length (Figure S5C in Supplementary Material) of neonates did not differ between treatment groups. These results suggest that neutrophils played a protective role in pregnancy in nSP70-treated mice—particularly in the maintenance of pregnancy.

### Neutrophil Depletion Leads to Placental Dysfunction in nSP70-Treated Mice

**Figure 4:**
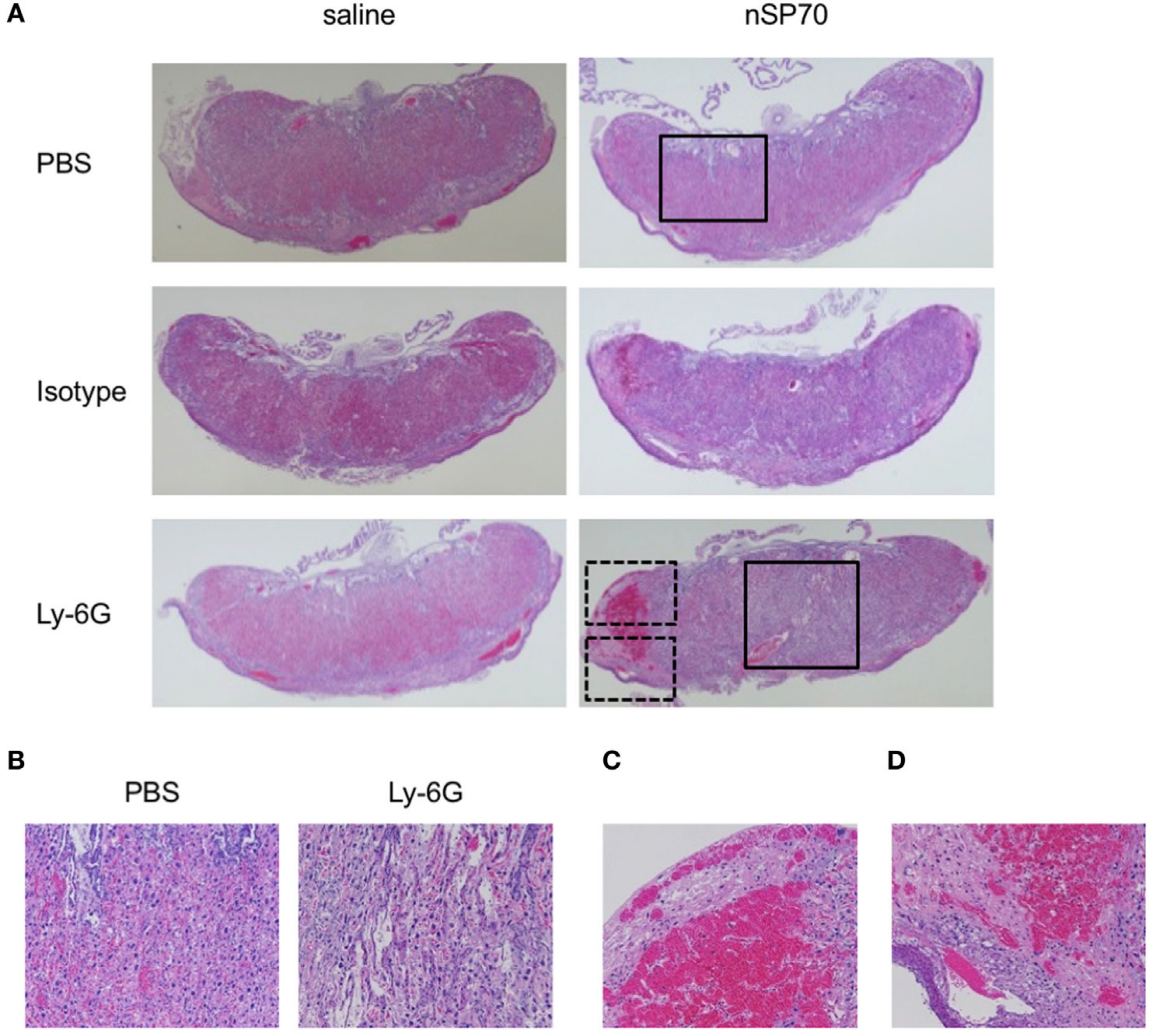
Structural abnormalities of placenta. Pregnant BALB/c mice were intraperitoneally injected with anti-Ly-6G or isotype-matched control antibodies (150 μg/mouse) on gestational day (GD) 15; 24 h later, mice received silica nanoparticles with a diameter of 70 nm (0.8 mg/mouse) or saline by intravenous injection. The placentae were excised on GD 17. **(A)** Placental sections were stained with hematoxylin and eosin and examined histologically. **(B)** Magnified images of the areas within the solid boxes in panel **(A)**. Magnified images of the areas within the **(C)** top and **(D)** bottom dashed boxes in panel **(A)**.

Normal placental development is required for embryonic growth, and placental dysfunction has been associated with miscarriage and fetal growth restriction (20, 21). Therefore, we considered that neutrophil depletion in nSP70-treated mice might alter placental function. To assess the relationship between neutrophil depletion and placental dysfunction, we histologically evaluated placental pathology by staining with hematoxylin and eosin (HE) (Figure 4A). The placentae of mice treated with nSP70 in the absence of neutrophil depletion showed tissue damage in the spongiotrophoblast layer, which segregates the maternal vasculature from the fetal vasculature in the placenta. In addition, blood flow was poor overall, and scant blood was present in the vessels around the decidua and chorionic plate. We obtained these same results in our previous study (5). In contrast, the placentae of nSP70-treated mice pretreated with anti-Ly-6G antibodies showed pronounced tissue damage from the decidua through the spongiotrophoblast layer, which now also contained areas of necrosis (Figure 4B). Furthermore, the placentae of nSP70-treated mice pretreated with anti-Ly-6G antibodies showed marginal congestion (Figure 4C) and bleeding in the decidua (Figure 4D). Comparison of the placentae of nSP70-treated mice with and without anti-Ly-6G antibody pretreatment revealed a slight decline in neutrophil abundance in the placentae of nSP70-treated mice pretreated with anti-Ly-6G antibody (Figure S6 in Supplementary Material). These results suggest that the exacerbation of pregnancy complications induced by peripheral neutrophil depletion is related to the decline in neutrophil abundance in the placental tissue.

**Figure 5:**
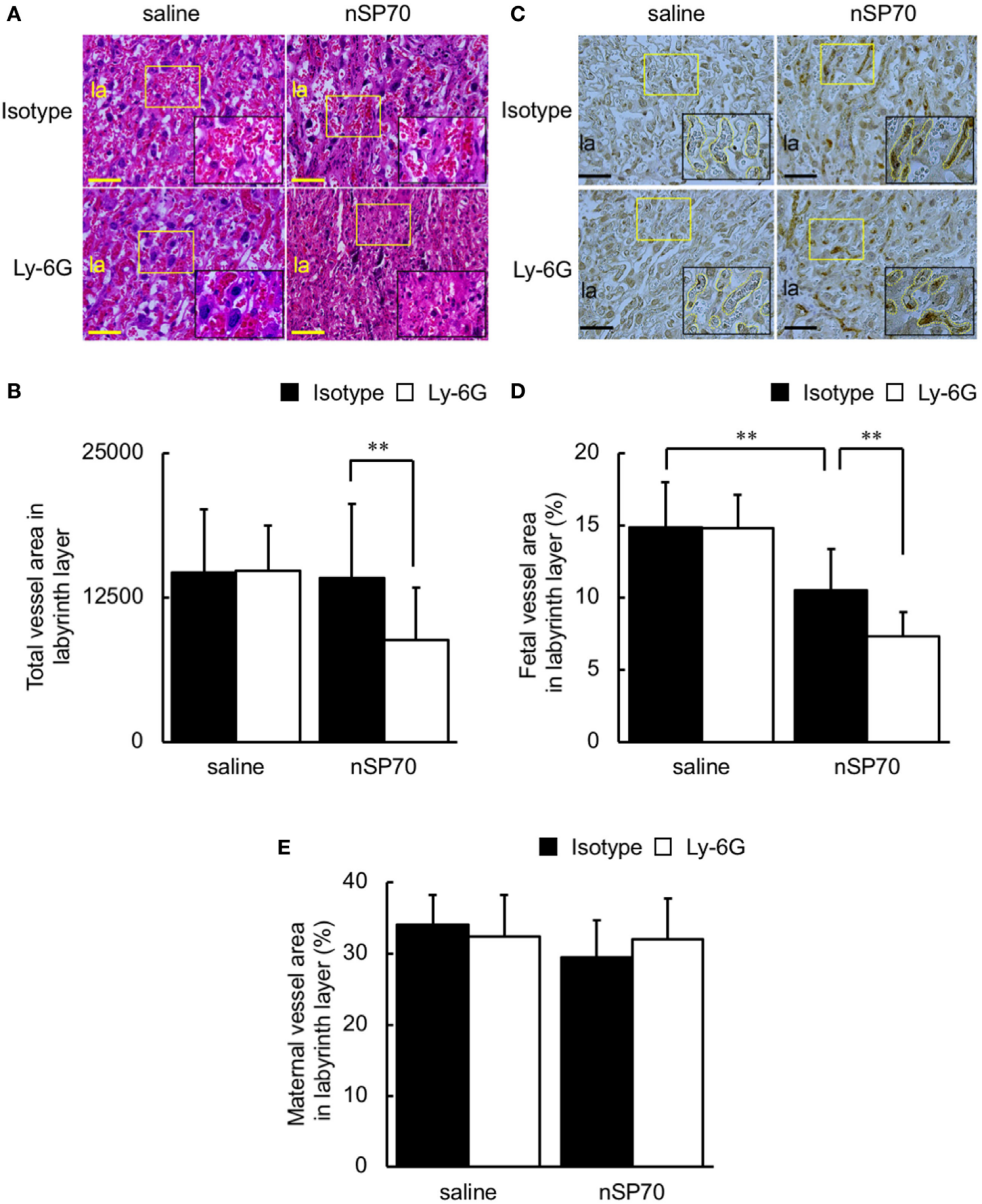
Neutrophil depletion decreases vessel area in the placentae of silica nanoparticles with a diameter of 70 nm (nSP70)-treated mice. Pregnant BALB/c mice were treated intraperitoneally with anti-Ly-6G or isotype-matched control antibodies (150 μg/mouse) on gestational day (GD) 15; 24 h later, mice were injected intravenously with nSP70 (0.8 mg/mouse) or saline. Placentae were excised on GD 17. **(A)** Placental sections were stained with hematoxylin and eosin and examined histologically. The area in the yellow rectangle was enlarged and is shown at lower right. **(B)** Several random areas were selected per placenta, and total vessel areas in the labyrinth layer were calculated. Data are presented as means ± SD; *n* = 34–62. **(C)** Placental sections were stained with CD31. The area in the yellow rectangle was enlarged and is shown at lower right. Fetal vessels are traced with yellow lines in the enlarged insets. Three regions per placenta were randomly selected, and the proportions of **(D)** fetal and **(E)** maternal vessel area to total area were calculated. Data are presented as means ± SD; *n* = 12–16; **P < 0.01. Scale bar: 100 µm. Abbreviation: la: labyrinth layer.

Because narrowing of placental vessels impairs placental formation (22), we histologically evaluated the area of spiral arteries, maternal vessels, and fetal vessels in the placentae. The spiral arteries, which delivery maternal blood to the labyrinth layer in the placenta (23, 24), were narrower in the placentae of anti-Ly-6G–nSP70-treated mice than in mice pretreated with isotype-matched control antibodies (Figures 5A,B). In addition, the placental structure in mice is hemochorial, and fetal, but not maternal, vessels are lined with endothelium (16). We, therefore, performed CD31 staining to differentiate fetal vessels, the inner surfaces of which are covered with CD31-positive endothelium, from maternal vessels, which are CD31-negative. Scattered placental vessels were lined with CD31-positive cells in the absence of nSP70 treatment. In contrast, nSP70 treatment led to the presence of dense CD31-positive, narrow fetal vessels in the placentae (Figure 5C), and fetal vessel area was significantly smaller in the placentae of mice treated with nSP70 in the absence of anti-Ly-6G antibodies than in mice treated with saline (Figure 5D). In addition, anti-Ly-6G antibodies augmented the narrowing of fetal vessels in the placentae of nSP70-treated mice, but anti-Ly-6G–saline-treatment did not confer any effects on the fetal vessels (Figure 5D). Furthermore, neither nSP70 nor anti-Ly-6G antibodies impaired the maternal vessels in the labyrinth layer (Figure 5E). Taken together, these findings suggest that nSP70 impaired spiral arteries and fetal vessels, which were coated with endothelium appropriately, and these effects were augmented by neutrophil depletion.

**Figure 6:**
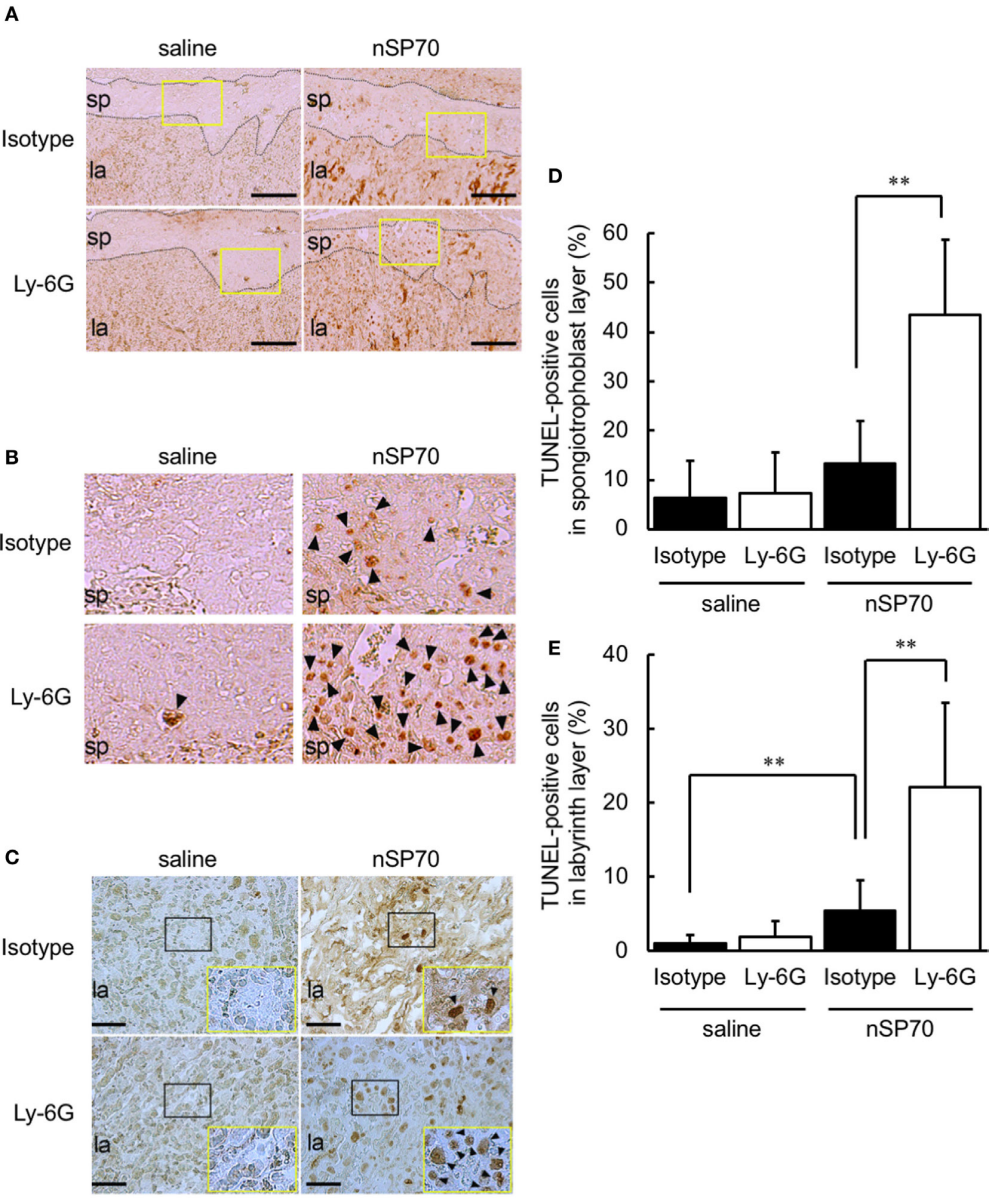
Treatment with silica nanoparticles with a diameter of 70 nm (nSP70) after neutrophil depletion increases the number of apoptotic cells in the placenta. Pregnant BALB/c mice were treated intraperitoneally with anti-Ly-6G or isotype-matched control antibodies (150 μg/mouse) on gestational day (GD) 15; 24 h later, mice were injected intravenously with nSP70 (0.8 mg/mouse) or saline. Placentae were excised on GD 17. Placental sections were stained with transferase-mediated dUTP nick-end labeling (TUNEL) to reveal apoptotic cells in the spongiotrophoblast and labyrinth layers. **(A)** The black dotted lines separate the spongiotrophoblast layer from the labyrinth layer. Scale bar: 200 µm. **(B)** The regions within the yellow rectangles in **(A)** are enlarged. **(C)** In the labyrinth layers, the regions within the black rectangles are enlarged and are shown at lower right. Scale bar: 100 µm. TUNEL-positive nuclei in the **(D)** spongiotrophoblast layer and **(E)** labyrinth layer of placentae was counted; the apoptotic index for each section was calculated as the number of TUNEL-positive nuclei divided by the total number of nuclei within the evaluated section and converted to a percentage. Data are presented as means ± SD; *n* = 8–16; **P < 0.01. Arrowheads: TUNEL-positive cells. Abbreviations: la: labyrinth layer, sp: spongiotrophoblast layer.

Staining by using terminal TUNEL showed that apoptotic cells were more numerous in both the spongiotrophoblast layer (Figures 6A,B) and labyrinth layer (Figure 6C) of anti-Ly-6G–nSP70-treated mice than in mice treated with isotype-matched control antibodies followed by nSP70. In addition, the percentage of TUNEL-stained nuclei was significantly higher within the spongiotrophoblast layer (Figure 6D) and labyrinth layer (Figure 6E) of anti-Ly-6G–nSP70-treated mice than in those from nSP70-treated mice pretreated with isotype-matched control antibodies. These findings suggest that neutrophil depletion increased nSP70-induced placental damage, with the induction of apoptosis and a potential reduction of the number of fetuses.

## Discussion

Several epidemiological studies have demonstrated the association between maternal exposure to fine particles during pregnancy and adverse pregnancy outcomes, such as low birth weight, preterm birth, and fetal death (25, 26). For example, a cohort study in Ohio reported that women exposed to higher than the standard exposure level of PM_2.5_ (airborne fine particulate matter measuring <2.5 μm in diameter) over the course of pregnancy were at 19% increased risk for preterm birth (27). Although these reports support the adverse effects of maternal exposure to particulate air pollution on birth outcome, the mechanisms for these effects have remained unclear. Moreover, several studies have shown that some nanoparticles can induce pregnancy complications in mice, but the details of the mechanism are not completely understood.

From this viewpoint, here we sought to assess nanoparticle-induced reproductive toxicity in mice and to elucidate its mechanism. We found that the maternal body weights of nSP70-treated mice were decreased and that their placentae demonstrated tissue destruction in the spongiotrophoblast layer on GD 17, unlike those of saline-treated mice, as previously reported (5). In addition, neutrophil depletion exacerbated nSP70-induced pregnancy complications and placental damage (Figures 2 and 4), suggesting that neutrophils help to suppress pregnancy complications. Furthermore, our studies have demonstrated that, compared with controls, mice that received nSP70 had about 20% lower uterine weights and significantly higher fetal resorption rates. In contrast, although we confirmed the nSP70-induced decrease in maternal body weight, it was less pronounced than that seen previously, and fetal resorption rates here were lower than in those in our previous study. We consider that this difference might reflect the difference in the number of nSP70 injections between the two studies.

In this regard, our previous study evaluated the effects of silica nanoparticles on fetuses after two injections (5), whereas here we assessed the effects after a single injection of silica nanoparticles. In the previous study, we administered silica nanoparticles to pregnant mice twice without neutrophil depletion and evaluated the subsequent pregnancy complications. The maternal body weights of these twice-injected mice were lower than those of nSP70-treated mice with neutrophil depletion. However, almost all of the fetuses of the dams that received two injections died *in utero* (data not shown), and we surmised that it might be difficult to assess the effects of neutrophils on fetuses or placenta by using our previous procedure. We, therefore, elected to use a single injection of silica nanoparticles in the current study, and this difference in methodology might have caused the differences between the previous and present data.

It is important to note the differences in timing of the experimental treatments in the current study. Previously, we investigated the time course of the change in the proportion of neutrophils after treatment with nSP70 in nonpregnant mice. Although the proportion in mice treated with nSP70 was significantly higher than that in saline-treated mice at 24 h, the proportions of neutrophils at both 2 and 72 h after nSP70 treatment were significantly lower than those in saline-treated mice at the same time points (15). We detected nSP70 in the livers of mice within 2 h of injection (28); therefore, prompt recruitment of neutrophils to the liver might have resulted in the transient decrease in the observed proportion of neutrophils in the peripheral blood 2 h after administration of nSP70. During the neutrophil’s lifespan of a few days, essential processes involved in restoring homeostasis after nSP70-induced neutrophilia may have resulted in the decrease in the proportion of neutrophils observed 72 h after nSP70 injection. Here, our treatments occurred relatively late in gestation, whereas some reports have studied similar effects earlier in pregnancy in the mouse. Girardi et al. (29) demonstrated that complement C5a-mediated recruitment of neutrophils in the placenta at day 8 of pregnancy is critical to pregnancy loss and the development of fetal damage. Nadkarni et al. (30) showed that, at a time of active placental development in the mouse, neutrophil-induced T-cells might be essential for normal placentation, including placental vascular development, and for fetal growth. Thus, there is a need to assess the effects of neutrophil depletion and nSP70 treatment not only in late pregnancy but also in early pregnancy.

Recent reports indicate that neutrophils may contribute to the clearance of nanoparticles. For example, using flow cytometry, Stephen et al. demonstrated that nanoparticles in both the peripheral blood and spleen were taken up at dramatically higher rates by granulocytes than by monocytes and that neutrophil depletion increased the numbers of particles in the blood (31). In addition, as shown in several recent studies (including our own), transportation of nanoparticles through the blood–placenta barrier is one of the causes of the induction of pregnancy complications by nanoparticles (32, 33). These combined results prompted our hypothesis that depletion of neutrophils, which take up nanoparticles, might increase the content of silica nanoparticles in the blood. Consequently, the distribution of silica nanoparticles to the placenta would increase as well, perhaps resulting in nSP70-induced structural abnormalities of the placenta. In this regard, the activated neutrophil population (CD16^bright^/CD62L^dim^) tended to be lower in nSP70-injected mice pretreated with anti-Ly-6G antibodies than in nSP70-injected mice pretreated with PBS or isotype-matched control antibodies (Figure S2 in Supplementary Material). Neutrophil activation may stimulate particle clearance; therefore, we consider that these results support our hypothesis that a decrease in the activated neutrophil population may lead to an increase in the translocation of silica nanoparticles from blood to placenta. Moreover, together with our histopathologic findings (Figure 4), the slight decline in neutrophil abundance in the placentae of nSP70-treated mice pretreated with anti-Ly-6G antibody (Figure S6 in Supplementary Material) suggests that the exacerbation of pregnancy complications by peripheral neutrophil depletion is related to a reduction in neutrophil abundance in the placental tissue and thus diminution of the protective effects on circulatory and placental tissues. Furthermore, to confirm our hypothesis, we sought to assess the silicon content of the placentae of mice after treatment with nSP70 with or without prior depletion of neutrophils. However, under both conditions the placental silicon concentration was below the limit of detection by inductively coupled plasma–atomic emission spectrometry (data not shown). Therefore, additional studies are needed to assess the changes in silica nanoparticle content in the placentae and blood of mice in which neutrophil counts are normal or depleted.

Here, HE staining and immunohistochemistry analysis revealed that, when neutrophil counts were diminished, nSP70 induced placental damage (Figure 4A), impaired placental vessels (Figure 5B), and increased the number of apoptotic cells in the placentae (Figure 6) compared with those under normal neutrophil counts. Normal placental development requires the coordinated production of vascular endothelial growth factor (VEGF) and its receptor, fms-like tyrosine kinase-1 (34), and neutrophil-derived VEGF-A induces the angiogenic activity of CXC chemokines (35), However, the production level of VEGF did not differ between the placentae of nSP70-treated mice pretreated with anti-Ly-6G antibodies and those of mice pretreated with isotype-matched control antibodies (data not shown). From this perspective, we propose that, once neutrophil depletion has increased the concentration of silica nanoparticles in the blood, the translocation of silica nanoparticles from blood to placenta increases. Thus, the cytotoxicity of nSP70 on placentae, including the vascular endothelium of spiral arteries and fetal vessels, might be exacerbated. However, the precise mechanism underlying nSP70-induced pregnancy complications remains to be clarified.

Preeclampsia and fetal growth restriction is known as complications associated with pregnancy, and involvement with autophagy has been reported as one of the causes (36). Autophagy is essential for vascular remodeling during the first stage of placentation (37), and impaired autophagy during preeclampsia increases the exposure of trophoblasts to oxidative and inflammatory stress (38). In addition, autophagy might suppress the activation of endotoxin-induced inflammasomes and may moderate the production of inflammatory cytokines, including interleukin (IL) 1β and IL-18 (36, 39). Inflammation in pregnant mice is thought to be an important factor in nanoparticle-induced pregnancy complications. Therefore, we considered that neutrophil depletion might disrupt autophagy, resulting in the activation of inflammasomes. Ultimately, neutrophil depletion may worsen the placental cellular damage induced by nSP70.

We demonstrated here that neutrophil depletion exacerbated pregnancy complications in nSP70-treated mice (Figures 2 and 3) and increased the placental damage induced by nSP70 (Figure 4). Although neutrophils are considered typical immunocompetent cells that promote inflammatory responses, it has recently been reported that neutrophils might directly contribute to the suppression of inflammation (40, 41). For example, neutrophil extracellular traps (NETs), resulting from excessive accumulations of neutrophils, reportedly induce the degradation of inflammatory mediators, including cytokines and chemokines, which are released by activated neutrophils; in this way they may terminate inflammatory responses (42). We previously reported that the concentrations of double-stranded DNA—a major component of NETs—in nSP70-treated mice were significantly higher than those in control mice (15). Therefore, we surmise that the neutrophil depletion that exacerbates pregnancy complications in nSP70-treated mice is connected with the protective roles of these cells, such as their contribution to the formation of NETs.

## Ethics Statement

All of the experiments involving mice were performed in accordance with the animal welfare guidelines of Osaka University and the National Institutes of Biomedical Innovation, Health, and Nutrition of Japan.

## Author Contributions

KH, YI, and YY designed the study. KH, AN, YI, AA, IY, and YL performed the experiments and analyzed data. KH, AN, and YY wrote the manuscript. AN, MN, IY, KN, ST, and SS provided technical support and conceptual advice. YT supervised all of the projects. All authors discussed the results and commented on the manuscript.

## Conflict of Interest Statement

YY is employed by the Research Foundation for Microbial Diseases of Osaka University. All other authors declare no competing financial interests.

## Abbreviations

FSC, forward-scattered light; HE, hematoxylin and eosin; IL, interleukin; NET, neutrophil extracellular trap; nSP70, silica nanoparticles with a diameter of 70 nm; PBS, phosphate-buffered saline; SSC, side-scattered light; TUNEL, TdT-mediated dUTP nick-end labeling; VEGF, vascular endothelial growth factor.
