# Supplementary material for: Neutrophil Depletion Exacerbates Pregnancy Complications, Including Placental Damage, Induced by Silica Nanoparticles in Mice
Source: Front Immunol. 2018 Aug 8;9:1850. doi: 10.3389/fimmu.2018.01850 (PMC6092495; doi:10.3389/fimmu.2018.01850)
Supplement: Supplementary file 1 [file data_sheet_1.PDF]

MATERIALS AND METHODS

Flow cytometry

To evaluate neutrophil subsets, cells collected from blood samples were labeled with combinations of phycoerythrin-conjugated Gr-1 antibodies (clone RB6-8C5, eBioscience), allophycocyanin-conjugated CD11b antibodies (clone M1/70, BD Pharmingen), fluorescein-isothiocyanate-conjugated CD16/32 antibodies (clone 2.4G2, BD Pharmingen), and phycoerythrin–Cy7-conjugated CD62L antibodies (clone MEL-14, BD Pharmingen). The different neutrophil subsets were analyzed for surface phenotype by means of flow cytometry (FACS Aria, BD Biosciences). Cells were gated according to side-scatter (SSC) area and forward scatter (FSC) area and then according to SSC height/SSC width, FSC height/FSC width, and 7-amino-actinomycin D staining to eliminate doublet cells and dead cells. Cells were gated according to $CD11b^{+}$ $Gr\text{-}1^{+}$ and then according to CD16/CD62L staining. Percentages of the total cell count were then analyzed. For analyses of $CD4^{+}$ T-cells and $CD4^{+}$ $FOXP3^{+}$ regulatory T-cells in the peripheral blood, cells collected from blood samples were labeled with phycoerythrin–Cy7-conjugated CD4 antibodies (clone RM4-5, eBioscience). After surface staining, the cells were fixed and permeabilized in accordance with the manufacturer's instructions (BD Pharmingen). Cells were stained intracellularly for FOXP3 (clone MF23, BD Pharmingen).

Hematology

Whole-blood samples were analyzed by using an automated hematology system (VetScan HMII, Abaxis, Sunnyvale, CA) to determine numbers of granulocytes, platelets, monocytes, lymphocytes, leukocytes, and erythrocytes.

Immunohistochemistry

Frozen placental sections (thickness, approximately 20 μm) were fixed in cold acetone for 8 min and then blocked with 20% fetal bovine serum in PBS for 1 h. The blocked tissue sections were stained with anti-mouse Ly-6G antibodies (1:500; clone 1A8, LEAF Purified, BioLegend) or isotype controls (1:500; clone RTK2758, BioLegend) in PBS containing 12% bovine serum albumin (Sigma-Aldrich, St Louis, MO). The sections were washed in PBS and stained at room temperature for 1 h with secondary antibodies (1:250; Alexa Fluor 594 goat anti-rat IgG antibodies, Invitrogen, Carlsbad, CA) in PBS containing 14% bovine serum albumin. Finally, the sections were washed in PBS and mounted by using an antifade reagent (ProLong Gold with DAPI, Thermo Fisher Scientific, Waltham, MA). Images were visualized by using a fluorescence microscope (model BZ-X710, Keyence, Osaka, Japan).

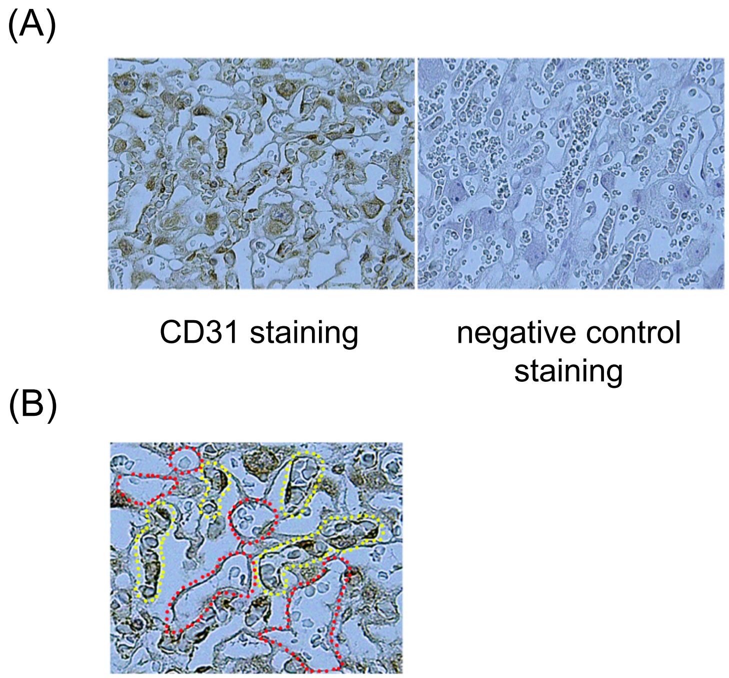

Supplementary Figure S1. CD31 staining in the labyrinth layer. (A) CD31 staining was performed to differentiate fetal vessels from maternal vessels. (B) Fetal vessels (outlined with yellow dotted lines) were lined with CD31-positive endothelium, but maternal vessels (outlined with red dotted lines) were not. Both vessels contained erythrocytes.

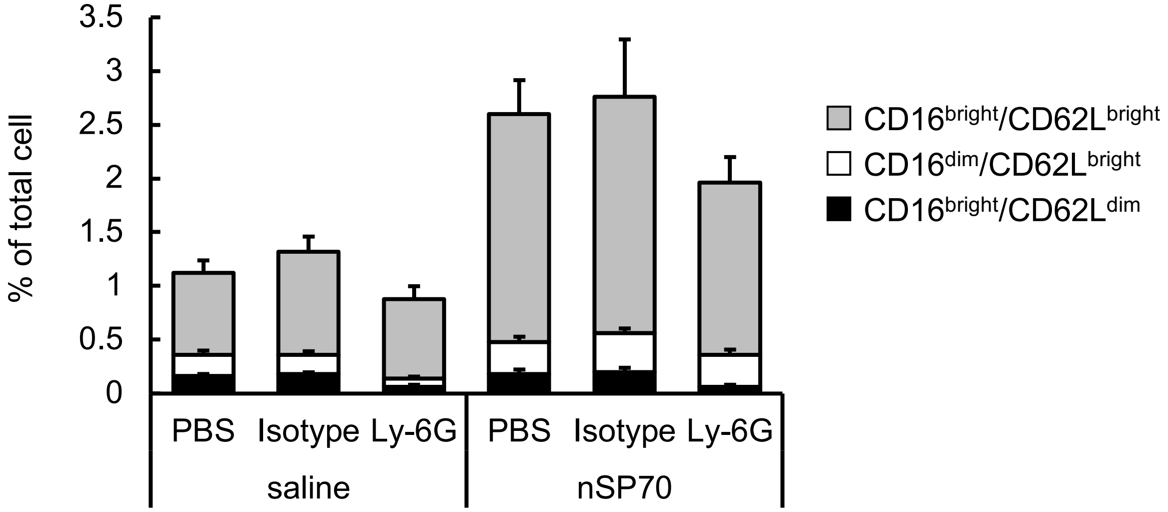

Supplementary Figure S2. Neutrophil subsets in pregnant mice after treatment with anti-Ly-6G antibodies. Pregnant BALB/c mice were intraperitoneally treated with anti-Ly-6G antibodies or isotype control antibodies (150 μg/mouse) on gestational day (GD) 15; twenty-four hours later, they received nSP70 (0.8 mg/mouse) or saline by intravenous injection. At 24 h after treatment, different neutrophil subsets were identified on the basis of CD16 and CD62L expression by using flow cytometry. Data are presented as means ± SEM; n = 5.

Figure S2. Higashisaka et al.

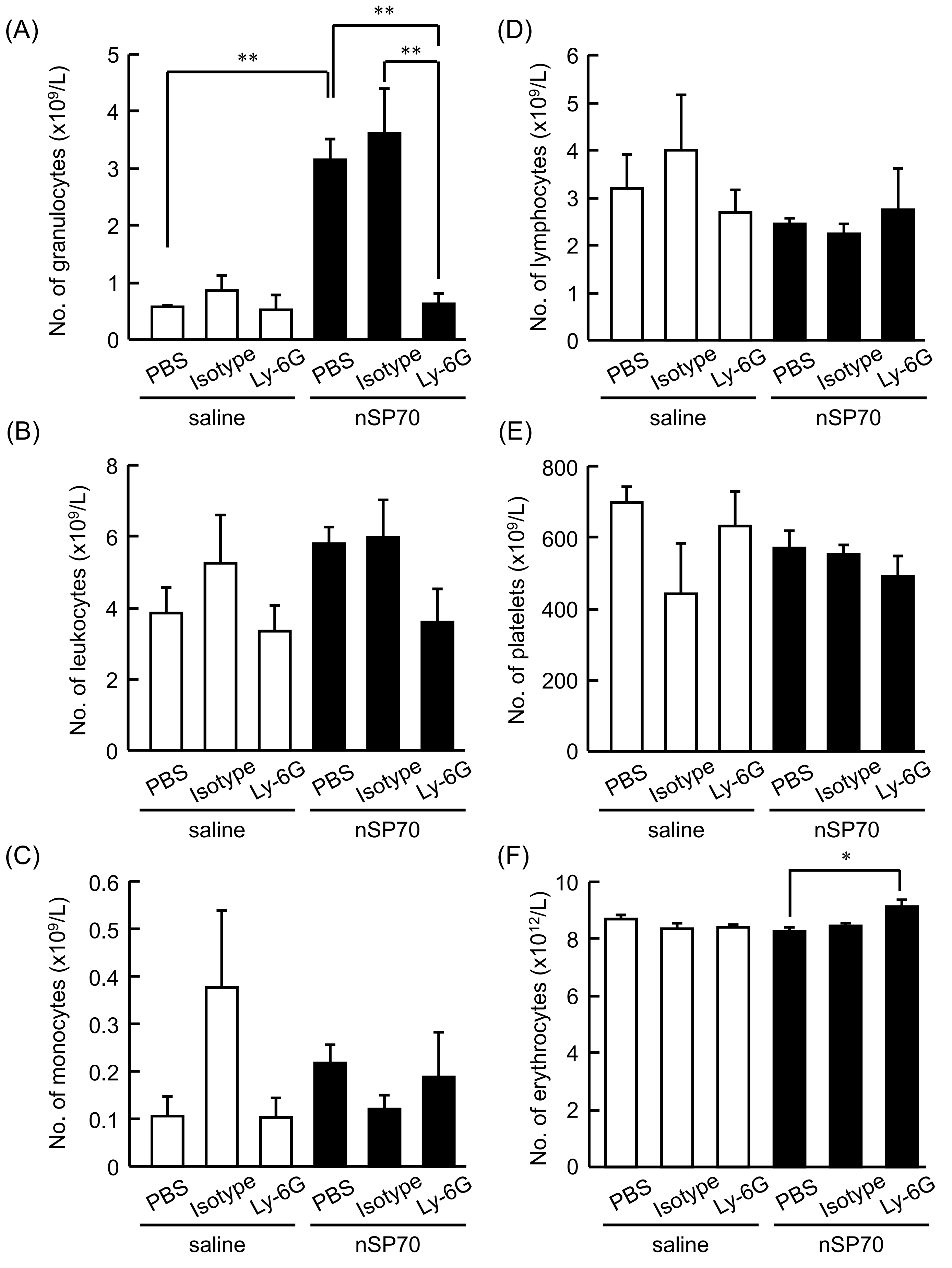

Figure S3. Higashisaka et al.

Supplementary Figure S3. Effects of treatment with anti-Ly-6G antibodies on blood cell counts in pregnant mice. Pregnant BALB/c mice were injected intraperitoneally with anti-Ly-6G antibodies or isotype-matched control antibodies (150 μg/mouse) on gestational day 15; twenty-four hours later, mice received nSP70 (0.8 mg/mouse) or saline by intravenous injection. Whole blood was collected from mice on gestational day 17. (A) Granulocytes, (B) leukocytes, (C) monocytes, (D) lymphocytes, (E) platelets, and (F) erythrocytes were counted. Data are presented as means ± SEM; n = 5 or 6; *$P < 0.05$, **$P < 0.01$.

(A)

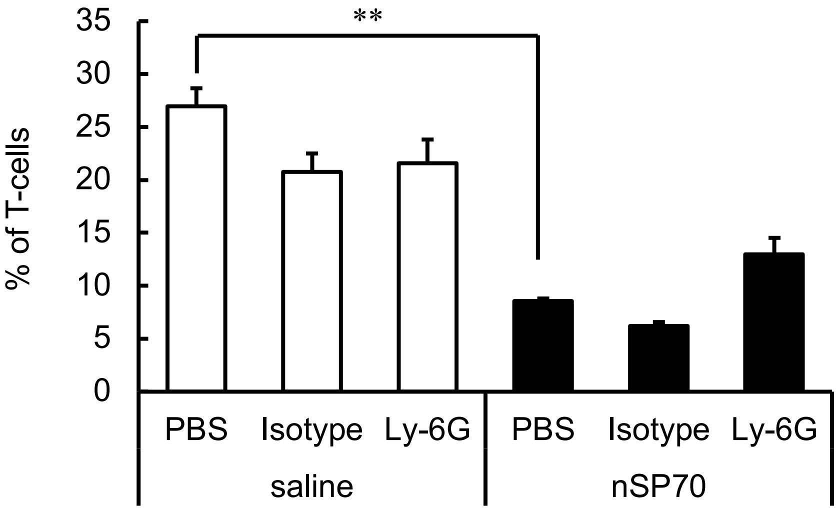

(B)

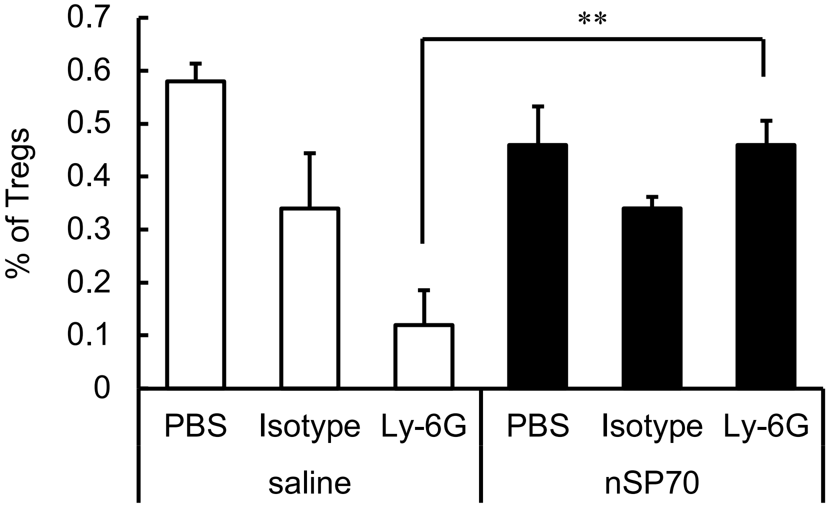

Supplementary Figure S4. Effects of pretreatment with anti-Ly-6G antibodies on T-cell levals in pregnant mice. Pregnant BALB/c mice were injected intraperitoneally with anti-Ly-6G antibodies or isotype-matched control antibodies (150 μg/mouse) on gestational day 15; 24 h later, mice received nSP70 (0.8 mg/mouse) or saline by intravenous injection. Percentages of (A) $CD4^{+}$ T-cells and (B) $CD4^{+}$ $FOXP3^{+}$ regulatory T-cells (Tregs) in peripheral blood were determined by using flow cytometry. Data are presented as means ± SEM; n = 5; $^{**}P < 0.01$.

Figure S4. Higashisaka et al.

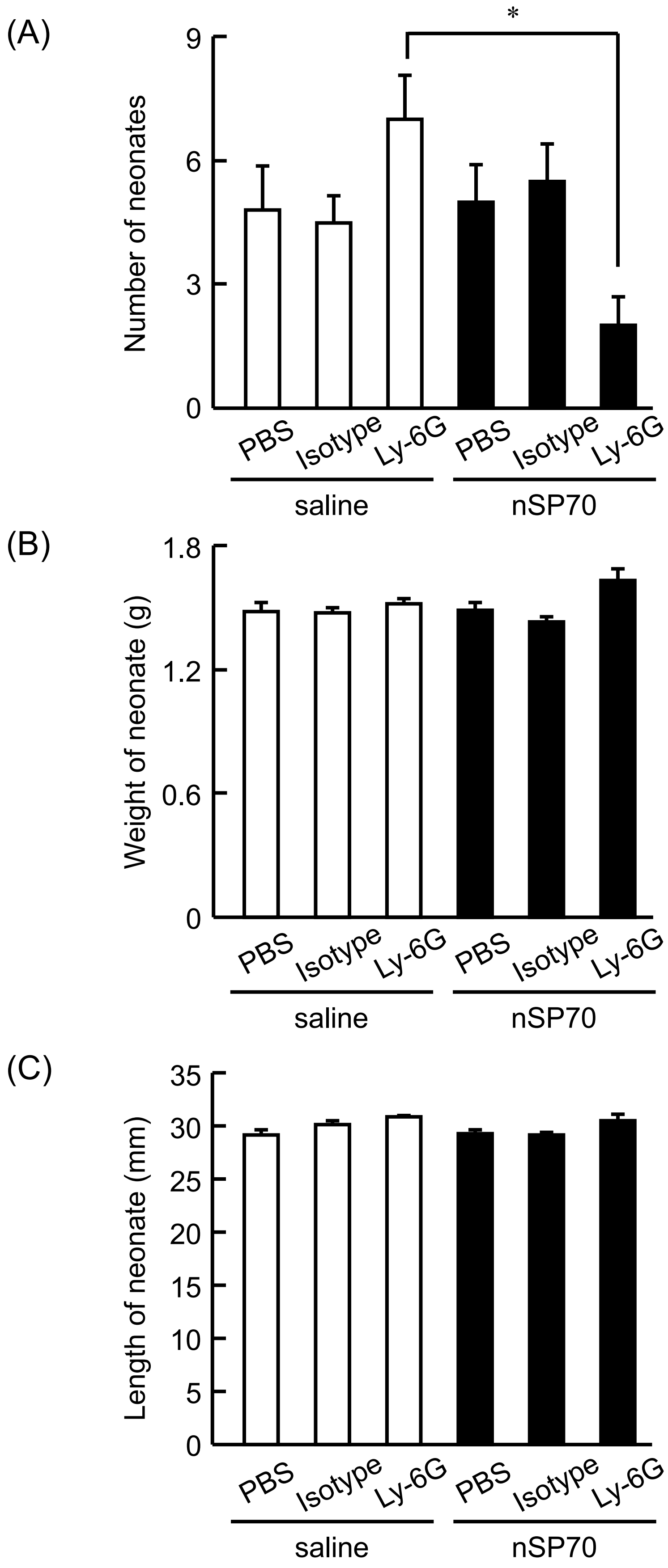

Figure S5. Higashisaka et al.

Supplementary Figure S5. Neutrophil depletion decreases the number of neonates from nSP70-treated mice. Pregnant BALB/c mice were intraperitoneally injected with anti-Ly-6G or isotype-matched control antibodies (150 μg/mouse) on gestational day 15; twenty-four hours later, mice received nSP70 (0.8 mg/mouse) or saline by intravenous injection. (A) Mice were monitored, and live-born neonates were counted. (B) The weight and (C) length of neonates were measured. Data are presented as means ± SEM; $n = 5$ or 6; $^*P < 0.05$.

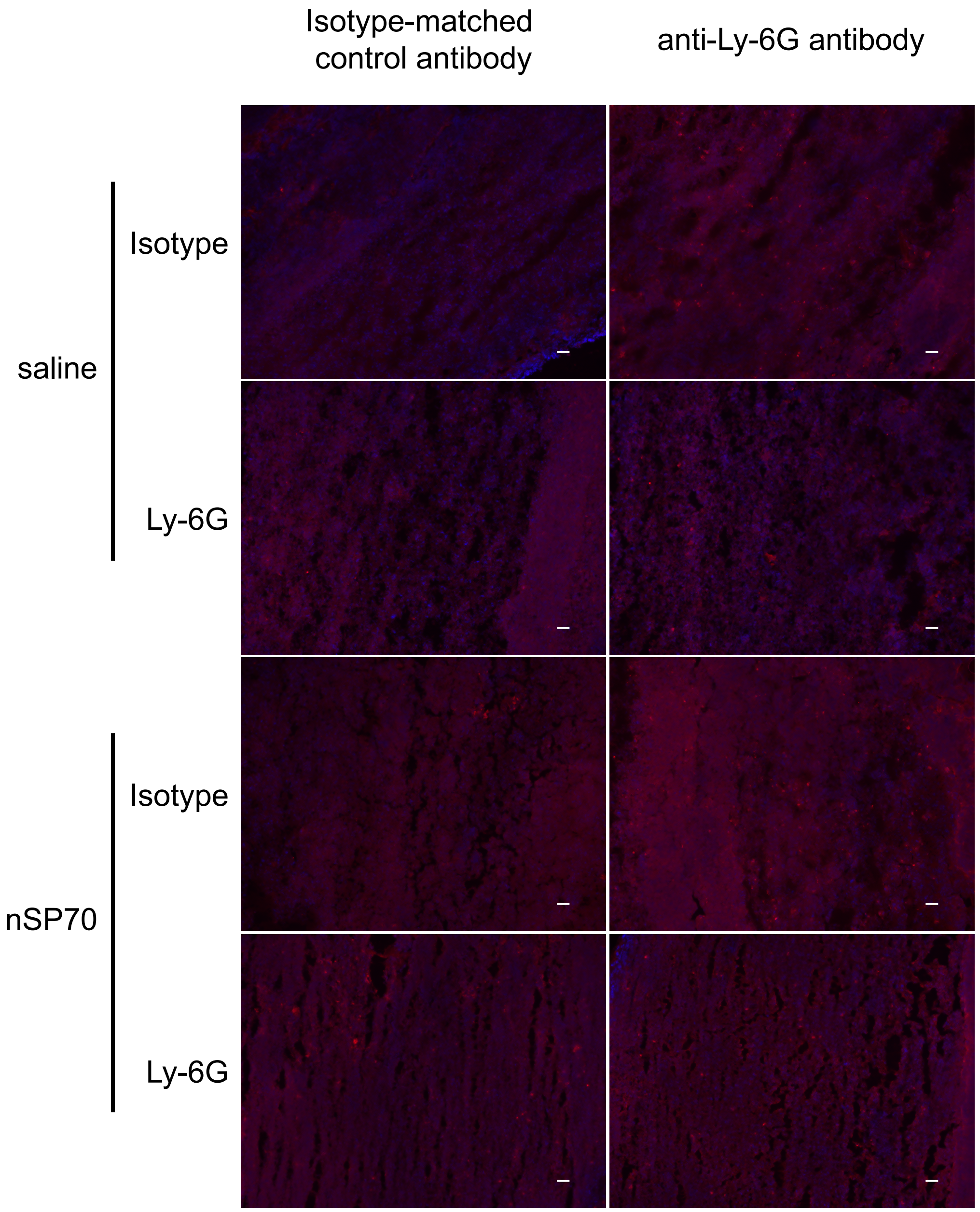

Supplementary Figure S6. Fluorescence microscopy images of mouse placentae after treatment of mice with silica nanoparticles. Pregnant BALB/c mice were intraperitoneally injected with anti-Ly-6G or isotype-matched control antibodies (150 μg/mouse) on gestational day 15; 24 h later, mice received nSP70 (0.8 mg/mouse) or saline by intravenous injection. Sections of placentae were stained with DAPI (blue), anti-Ly-6G antibodies (red), or isotype-matched control antibodies and were visualized by using a fluorescence microscope (BZ-X710, Keyence). Scale bar, 50 μm.

Figure S6. Higashisaka et al.
